# Initial implicit association between whole grains and taste does not predict consumption of whole grains in low-whole grain consumers: a pilot randomized controlled trial

**DOI:** 10.3389/fnut.2024.1408256

**Published:** 2024-09-30

**Authors:** Angela De Leon, Dustin J. Burnett, Bret Rust, Marika Lyly, Nancy L. Keim

**Affiliations:** ^1^Graduate Group in Nutritional Biology, University of California, Davis, Davis, CA, United States; ^2^Western Human Nutrition Research Center, Agricultural Research Service (USDA), Davis, CA, United States; ^3^VTT Technical Research Centre of Finland Ltd., Espoo, Finland; ^4^Lantmannen Unibake Finland Fresh (VAASAN Oy), Helsinki, Finland

**Keywords:** implicit association, Implicit Association Test, mere exposure, taste, whole grain consumption, whole grains

## Abstract

**Background:**

Health benefits of whole grain (WG) consumption are well documented. Current Dietary Guidelines for Americans recommend at least half of total grains consumed be WG; however, Americans consume less than one serving of WG per day. Inferior taste of whole grain products as compared with refined grain products has been reported as one of the main barriers to acceptability and consumption of whole grains. In this pilot study, we aimed to determine if mere exposure to WG foods in self-reported low WG consumers would improve their implicit associations between WG and pleasant taste.

**Methods:**

Healthy adults (*n*=45) were provided a variety of WG or refined grain (RG) products for home use for 6 weeks. Intake was measured by calculating disappearance and verified by a daily log. At the beginning and end of the intervention, we administered an Implicit Association Test (IAT), a computer test designed to measure indirectly the strength of association between pairs of concepts: (a) two contrasted target categories (WG and RG food images) and (b) two contrasted attribute categories (words relating to pleasant or unpleasant taste) via a classification task. Response time was used to calculate IAT D scores, indicating the strength of implicit associations between WG and RG and positive or negative taste.

**Results:**

ANCOVA showed that average D scores at the end of the study shifted significantly toward a positive implicit association between WG and good taste (*p*<0.05) in participants whose baseline D scores indicated an initial preference for RG over WG. No significant differences were found between the WG and RG groups in overall consumption of provided grain products.

**Conclusion:**

These findings suggest that mere exposure to WG products over an extended period of time in a free-living situation can improve automatic attitudes toward WG, potentially leading to increased consumption of WG foods.

**Clinical trial registration:**

Clinicaltrials.Gov, identifier NCT01403857.

## 1 Introduction

The health benefits of whole grain (WG) consumption are well documented (1–3). Whole grain consumption has been associated with reduced risk of cardiovascular disease (4, 5), type 2 diabetes (6, 7), inflammation (8), and certain cancers (9–12). In addition, large prospective studies of the US population found higher whole grain consumption associated with lower total mortality (13–15). Accordingly, since 2005, the Dietary Guidelines for Americans (DGA), recommend that individuals consume ≥3 ounce-equivalents/servings of WG/day or that at least half of total grains consumed be WG (16). The food industry has responded to these guidelines with a dramatic increase in the production and marketing of WG products, along with efforts to assist the consumer in identifying WG products at the point of purchase (17, 18).

Despite increased variety, availability, and promotion of WG products, consumption of WG in the US remains low, with Americans in all age groups still consuming less than one serving of WG per day (19–21). Reported barriers to WG consumption include inferior taste and texture in comparison to RG products, cost, availability, convenience, and lack of knowledge of the health benefits of WG (22, 23).

Research suggests that much of human behavior is driven not by conscious deliberation of immediate choices or concern over long term health outcomes, but rather by habit and other automatic processes that are extremely efficient (24–27). Numerous studies support this idea (28–30). Despite good dietary intentions, stress and increased cognitive load can impair an individual’s ability to choose healthy options when presented with highly palatable, calorie-dense foods (31–33).

In addition to scenarios in which eating occurs as an automatic action triggered by powerful environmental or situational cues, other implicit processes may contribute to eating behavior and food choice. Social psychologists Greenwald and Banaji describe implicit attitudes as “introspectively unidentified…traces of past experience that mediate favorable or unfavorable feeling, thought, or action toward social objects” which “manifest as actions or judgments that are under the control of automatically activated evaluation” (34). Just as individuals make automatic, unconscious evaluations that determine judgments and actions in social situations, implicit attitudes toward particular foods may predict food choice. Studies investigating the influence of implicit attitudes on consumer food choice behavior have demonstrated that in some cases automatic associations may play a larger role than self-reported motivators such as perceived nutritional value or potential health benefits (35–38). A better understanding of implicit processes in human dietary choices may contribute to designing effective interventions to improve dietary behaviors such as consuming enough WG foods to achieve optimal health benefits.

Measures that capture implicit associations are widely used in consumer psychology, organizational management, and marketing research, as these fields recognize the limitations of self-reported explicit measures (35, 37, 39–41). One of the most widely used measures of implicit attitudes in nutrition research is the Implicit Association Test (IAT) (42–44). The IAT is designed to measure strengths of associations between contrasting target concepts and contrasting attributes by measuring response times in a computerized sorting task. The assumption underlying the interpretation of the IAT is that responses will be faster and more accurate when the target and attribute categories are more strongly associated. The measure is described as implicit because it operates without the test taker’s awareness of the existence or strength of the associations under question.

The mere exposure paradigm consists of repeatedly exposing an individual to a novel or less liked stimulus object (45, 46). Although the individual is not required to engage in any kind of behavior or evaluation at the time of the exposure, it has been demonstrated that simply by mere exposure, acceptance and preference for the stimulus object can be enhanced (45–47). Research on acceptance of novel foods indicates that through repeated exposure, initially less palatable or unfamiliar foods ultimately achieve higher acceptance (48–51). If the mere exposure effect worked in our study, this would predict that people unfamiliar with WG would demonstrate increased liking and acceptability for these products after repeated exposures. Moreover, as implicit associations are rooted in experiences not consciously monitored or remembered, it is possible that mere exposure to WG foods in self-reported low WG consumers in this study could lead to a stronger implicit association between WG foods and pleasant taste.

The aims of the current study were to determine if: (1) initial implicit associations between WG/RG foods and taste (pleasant/unpleasant) predict consumption of provided WG products and (2) mere exposure to WG products incorporated into the daily diet of self-reported low WG consumers strengthens the association of WG foods with pleasant taste. If an implicit association between whole grain foods and pleasant taste can be strengthened by mere exposure, this may ultimately lead to consumers choosing whole grains.

## 2 Materials and methods

This pilot study was conducted in accordance with the ethical standards set by the University of California, Davis Office of Research Institutional Review Board (IRB ID 235561) and is registered at clinicaltrials.gov as NCT01403857. All participants provided written informed consent and received monetary compensation for their participation.

### 2.1 Participants

Healthy men and women, aged 20–45, with a body mass index between 18.5 and 32.0 kg/m^2^ and stable body weight (within ± 3 kg) for the previous 6 months were recruited from Davis, California and outlying areas. Eligible participants prepared and ate the majority of their meals at home, and their habitual consumption of whole grains was ≤ 1 serving/day based on self-report. During the screening visit for the study, participants filled out an extensive questionnaire designed to assess the typical level of WG consumption (52). Questionnaire items included specific questions regarding the type, amount, and frequency of consumption of all grain products on a daily, weekly, biweekly, and monthly basis. Participants agreed to incorporate provided study foods into their daily diet for the duration of the 6-week intervention. Participants also agreed to continue their usual physical activity practices. Exclusion criteria included: currently dieting to lose weight; pregnant currently or within the past 6 months; diagnosis of type 1 or 2 diabetes; gastrointestinal diseases; regular use of colonics or laxatives; recent (within 3 months) use of antibiotics, appetite suppressants or mood-altering medications; regular use of tobacco products.

### 2.2 Study design

Details of this study have been previously described (52, 53). In brief, this pilot study was a 6-week parallel arm intervention study where participants were randomly assigned in permuted block sizes of 3 in a 2:1 ratio of those receiving WG to those receiving refined grain (RG) products (53). The reason for this ratio was that we were particularly interested in precisely measuring outcome variables in the WG group. The RG group functioned as a control group. The WG and the RG groups received the assigned grain products in weekly allotments containing the recommended number of grain servings based on individual caloric needs for weight maintenance. The provided grain products could be incorporated into meals or eaten as snacks throughout the day. Participants randomized to the WG intervention received WG products representing commonly consumed grain products in the US. Participants in the RG intervention received closely matching RG versions of the same foods. For the WG group, the products approached 100% of recommended total grain servings per day as WG; for the RG group, no WG products were provided. Provided grain product consumption was tracked by weekly logbooks used by participants.

To evaluate the effects of the intervention on implicit associations between WG and RG and taste, the Grains IAT was administered during a baseline test day prior to initiating the intervention and again on a second test day during the sixth week of intervention.

### 2.3 Grain products

A variety of grain products were provided by the USDA Western Human Nutrition Research Center (WHNRC) on a weekly basis to participants for 6 weeks. Some of the grain products were formulated and prepared by the Metabolic Kitchen and Human Feeding Lab in the WHNRC; others were commercially available foods (52, 53). Products were weighed and packaged without brand identification or nutrition information to avoid bias. Participants randomized to the WG group received WG sliced bread, ready-to-eat breakfast cereal, crackers, rice, couscous, penne pasta, spaghetti, tortillas, cookies, cornbread muffins, and baking mix. Some of the foods were provided both dry and cooked (couscous and pastas). The baking mix only required water to use for preparing pancakes or muffins. Participants in the RG group received closely matching RG versions of the same foods. Grain products were packaged and labeled according to the instructions for home storage food safety: room temperature (ready-to-eat), refrigerator, or freezer. The number of grain servings provided was based on the number of servings recommended for each participant’s energy needs. For example, a participant with an estimated energy expenditure of 2000 calories per day would receive six servings of grains per day, for a total of 42 servings of grains per week. The estimation of resting energy expenditure for each participant was calculated using the Harris-Benedict equation (54), incorporating anthropometric data obtained during the screening visit. A light activity factor of 1.4 was used as a multiplier for resting energy expenditure to determine total daily energy expenditure.

### 2.4 Measuring exposure

In this study, mere exposure to WG was operationalized as provision of grain products during the 6-week intervention. Because all participants accepted into the study were self-reported low whole grain consumers, provision of WG to the WG group functioned as the mere exposure. Participants were instructed to record consumption of all grain products in a weekly log booklet, including grain products not included in study foods. Instruction was provided for recording accurate daily log entries, including details about preparation methods, amount, location, and time of day the grain products were consumed. Recipe suggestions, measurement aids, and blank notes pages were provided in the booklets. Participants returned unused and prepared-but-uneaten foods and all packaging materials along with the log booklet at the end of each week and then received the next week’s allotment of products. Total servings of grain products consumed were calculated by measuring the disappearance of provided foods and by analyzing data recorded in the weekly logs. Participants were not required to consume all of the grain products provided each week but were encouraged to incorporate the provided foods into their daily meals and snacks in place of the products they would normally purchase for themselves. Participants were also instructed not to share their study foods with others.

### 2.5 Procedures

#### 2.5.1 Test day protocol

Participants arrived on the morning of each test day after an overnight fast. After a short period for taking anthropometric and other study measures, they were given a standard light breakfast consisting of peach yogurt, apple slices, peanut butter, and bottled spring water. Approximately 45 min after completing breakfast, participants were escorted into a sound-proofed cognitive testing booth where they were seated in front of a desktop computer with a 17 in. flat screen monitor. The IAT task was administered using experiment generator software (Inquisit 3.0, Millisecond Software, Seattle, WA). Before starting the test, researchers read the on-screen instructions aloud and confirmed participants’ understanding of the task procedures. After starting up the test, the investigator exited the cognitive testing booth but remained in the general area in the case a participant had any questions or concerns during the testing. Participants were told that the purpose of the computer test was to gather information about their food preferences. Thus, they were unaware that their reaction speed was being measured.

#### 2.5.2 The implicit association test

The IAT Is a computerized test that indirectly measures the strength of association between pairs of concepts: (a) two contrasted target categories and (b) two contrasted attribute categories via a classification task. Stimuli for the four categories (Supplementary Table S1) in this study included: (1) photographic images of RG foods, (2) photographic images of WG foods, (3) words associated with tastiness or enjoyment of food (e.g., tasty, delicious), and (4) words associated with lack of taste or enjoyment (e.g., flavorless, unappealing). Participants were instructed to rapidly classify stimuli that represented target and attribute into one of four distinct categories as quickly and accurately as possible using only two response keys on the computer keyboard. The food image or taste word appeared in the center of the computer screen and stayed onscreen until the participant responded. In the case of an incorrect response (e.g., pressing the key for “Refined grain” for an image of a whole grain food), a red “X” appeared on the screen and the participant had to correct the response in order to continue. An inter-stimulus interval of 250 ms was used. Response latency was measured in milliseconds, providing a measure of the strength of association between target and attribute.

The IAT started with practice blocks in which only images of WG or RG foods or words relating to positive or negative taste appeared on the screen and participants classified them using the E or I key on the keyboard. Participants referred to descriptors positioned on the top left and right corners of the computer screen which indicated the correct response (Supplementary Figure S1). These descriptors remained on the screen for the duration of each practice and test block. The task became more complex in subsequent practice and test blocks where the categories were combined, and the participants had to sort a WG or RG grain food with a positive or negative taste word using only two keys. In the combined practice and test blocks, participants were instructed to use the same key for either WG or RG *and* positive or negative taste. The idea being that the stronger the implicit association between target and attribute, the faster the response time would be. In the second set of combined blocks the target-attribute combination was reversed. Table 1 lists the order of practice and test blocks, which is consistent with that described by the originators of the IAT (55, 56). For each participant session, the order of stimulus presentation was randomized within each practice and test block.

**Table 1:** Sequence of trial blocks used in the implicit attitude test^1^.

Block	Number of trials	Function	Items assigned to left key	Items assigned to right key
1	20	Practice	RG food	WG food
2	28	Practice	Positive taste	Negative taste
3	24	Practice	RG and positive taste	WG and negative taste
4	48	**Test**	**RG and positive taste**	**WG and negative taste**
5	40	Practice	Negative taste	Positive taste
6	24	Practice	RG and negative taste	WG and positive taste
7	48	**Test**	**RG and negative taste**	**WG and positive taste**

^1^RG, refined grain; WG, whole grain. The bolding is for emphasis of the differences and adds meaning to the understanding of the tests.

Response time measured in milliseconds was used to calculate an IAT *D* score averaged over all participants and for each individual participant, as recommended by Greenwald et al. (56). The *D* score is computed as the difference in average response time between the IAT’s two combined tasks (e.g., RG and good taste, WG and bad taste; WG and good taste, RG and bad taste), divided by a pooled standard deviation of participant response times in the two combined tasks (Table 2). The resultant statistic is an effect size similar to a Cohen’s *d* effect size, the main difference being that the standard deviation in the denominator of *D* is calculated from the scores in both conditions, whereas the Cohen’s *d* score is computed using a pooled within-treatment standard deviation (56). Using this algorithm, the resultant measure is the IAT *D* effect. This *D* score indicates overall implicit association between RG and WG foods and good or bad taste. We designed the computer program so that positive values of this score indicated faster reaction times in the ‘congruent’ blocks in which RG foods and pleasant taste shared the same response key, signifying a stronger implicit association between RG foods and pleasant taste, as compared to WG foods and pleasant taste; negative values indicated the reverse associations. Presentation order of the congruent and incongruent test blocks was counterbalanced among participants. A score of zero indicates no difference in preference for WG or RG foods.

**Table 2:** Summary of IAT scoring procedures recommended by Greenwald et al. (56).

1. Delete all trials greater than 10,000 milliseconds.
2. Exclude participants whose response times were less than 300 milliseconds on more than 10% of trials.
3. Compute one pooled standard deviation for all trials in Blocks 3 and 6; another for all trials in Blocks 4 and 7.
4. Compute the mean of correct latencies for each of Blocks 3, 4, 6, and 7.
5. Compute the two mean differences (Mean Block 6 – Mean Block 3) and (Mean Block 7 – Mean Block 4).
6. Divide each difference score by its associated pooled-trials standard deviation from step 3.
7. *D* score is the equal-weight average of the two resulting ratios in step 6.

## 3 Statistical analysis

Data were exported from Inquisit to Microsoft Excel for preparation and analyzed using SAS for Windows Release 9.4 (Cary, NC, United States). The number of weekly servings of grain products provided to participants varied according to caloric needs for weight maintenance, thus percentage of provided products consumed was used in the analysis to compare the difference in consumption between groups. Grain product consumption and *D* scores were assessed for conformance to the normal distribution using the Shapiro–Wilk test. None of the variables needed to be transformed as they were all normally distributed. We tested for outliers in the data, identified as >3 SD from the mean, but none were found. Baseline *D* score was included in models as a continuous variable. Participant characteristics at baseline were compared between groups with Wilcoxon tests and chi-square tests. Percentage of provided products consumed at week 1, percentage of products consumed averaged across all 6 weeks, and change in IAT *D*-scores were compared between groups with analysis of covariance (ANCOVA), controlling for initial *D* score or grain type (WG vs. RG) preference, age, and sex of the participant. Change in percentage of products consumed from week 1 to week 6 was compared between groups with two-sample *t*-test. Tests were two-sided and significance level was set at *p* < 0.05. Data are presented as means ± SEMs unless otherwise noted.

## 4 Results

### 4.1 Recruitment

**Figure 1:**
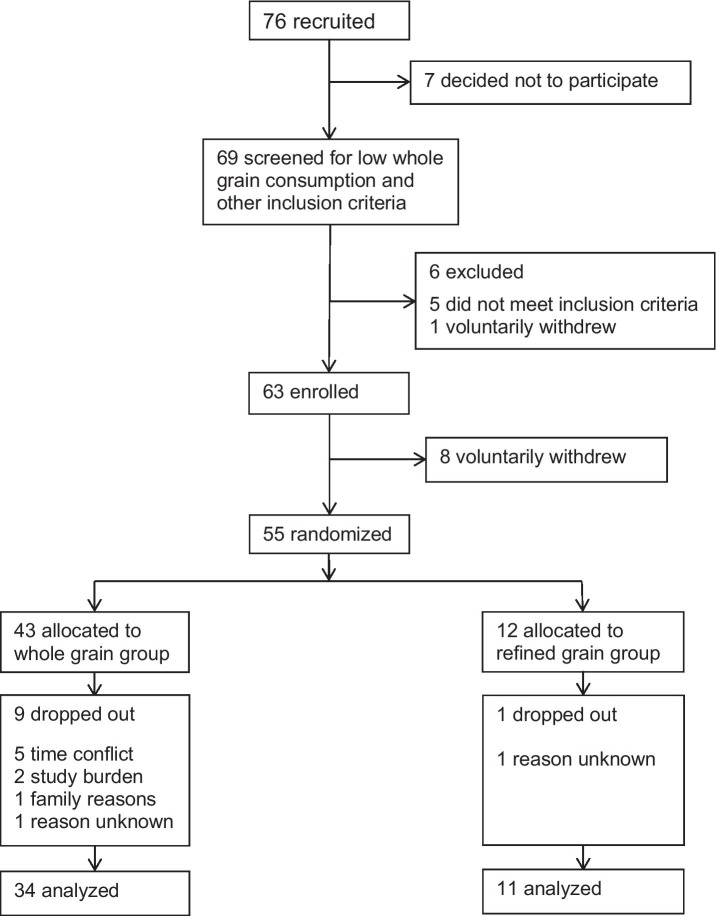
CONSORT flowchart of participants through the study.

The CONSORT flowchart of participants in this study is shown in Figure 1. Of the 63 participants who were enrolled, eight withdrew before randomization, leaving 55 participants to be randomized into either the WG or the RG intervention. Nine participants dropped out of the WG group for a variety of personal reasons; however, none stated dissatisfaction with study foods as the reason. One person in each group was lost to follow-up, no reason given, leaving 45 participants to complete the intervention. Although the randomization scheme was intended to result in a 2:1 ratio of WG to RG group sizes, the final number of completers totaled 34 in the WG group and 11 in the RG group, due in part to early termination of the study for funding reasons. Although the total number of participants was fewer than planned, the number of participants in the WG group exceeded the minimum required in our sample size calculation to be powered to measure changes in *D* scores, which was 30 participants for the WG group.

### 4.2 Participant characteristics

Of the 45 participants who completed the study, results from 43 are presented here. Technical difficulties prevented two participants from completing the computer IAT at the baseline visit. No significant differences were found between the whole grain and the refined grain group regarding age (*p* = 0.31), BMI (*p* = 0.35), or initial D score (*p* = 0.96), but there were significantly more female participants in the WG group than in the RG group (*p* = 0.043) (Table 3).

**Table 3:** Participant characteristics.

	Whole grain (*n* = 32)	Refined grain (*n* = 11)
Age (*y*)^2^	25.4 ± 6.0	24.2 ± 5.5
**Sex (n)**		
Male	12	8
Female	20	3
BMI (kg/m^2^)^2^	22.6 ± 2.6	25.6 ± 6.6
Initial *D* scores^3^	−0.175 ± 0.588	−0.156 ± 0.644
Prefer WG	21	7
Prefer RG	11	4

^1^BMI, body mass index; WG, whole grain; RG, refined grain.

^2^Values are means ± standard deviations.

^3^A negative D score indicates a preference for WG and a positive D score indicates a preference for RG.

Despite being self-reported low whole grains consumers, the majority of participants (28 out of 43, or 65%) had baseline IAT *D* scores indicating an implicit preference for whole grain foods over refined grain foods. This preference was well distributed between treatment groups. Participants who received the WG products consumed an average of 48 ± 3% of the foods provided over the 6-week intervention period, and those receiving the RG products consumed 45 ± 8% over the same time period.

### 4.3 Implicit taste preference and grain product consumption

**Figure 2:**
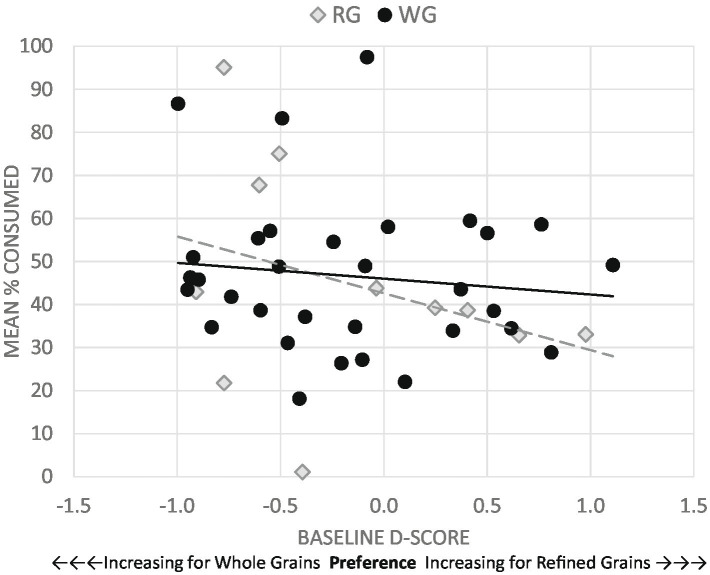
Percentage consumption averaged over the 6-week intervention for the whole grain (WG) and refined grain (RG) groups. Neither the WG group nor the RG group showed an association between the baseline D score and the percent of grain products consumed over the 6-week intervention.

The primary aim of this study was to determine if initial implicit taste preference for WG or RG would predict consumption of provided grain products. Thus, we examined the relationship between the initial *D* score and the percentage of provided grain products consumed during the first week of the study and again for all 6 weeks of the study. Within each treatment group, there was no association between consumption at week 1 and initial *D* score, WG foods (*r* = 0.242, *p* = 0.462) and RG foods (*r* = 0.012, *p* = 0.796). Combining the results of both treatment groups, there was also no association between consumption at week 1 and initial *D* score (*r* = 0.246, *p* = 0.990). Similarly, we found no association between initial *D* score and percentage of grain products consumed over the full 6-week period within each treatment group, WG foods (*r* = 0.197, *p* = 0.626) and RG foods (*r* = 0.424, *p* = 0.311), respectively, or when combining results of both treatment groups (*r* = 0.285, *p* = 0.162) (Figure 2).

Consumption of provided grain products did not change over time in participants who received the WG products. Further, we found that consumption of the WG products did not change from beginning to end of the intervention regardless of whether the participant preferred whole or refined grains initially (Table 4). Considering that participants in the WG consumed nearly half of the provided grain foods, this represents a significant increase in WG consumption in individuals who self-identified as consuming ≤1 serving of WG/day. However, for the participants who were assigned the RG foods, those who showed an initial preference for RG foods consumed less of the provided grain products at week 6 than at week 1 (*p* = 0.041) (Table 4).

**Table 4:** Consumption of grain products by participants according to initial preference^1^.

	Whole grain group		Refined grain group	
Initial implicit Preference	WG *n* = 21	RG *n* = 11	WG *n* = 7	RG *n* = 4

% of Grain products consumed^1^				
Week 1	49.2 ± 4.2	51.9 ± 4.0	43.3 ± 11.2	45.5 ± 5.0
Week 6	43.0 ± 5.0	40.8 ± 8.1	56.2 ± 13.2	31.7 ± 2.5
Change in % Wk1 – Wk6	−6.2 ± 4.5	−11.1 ± 10.0	13.0 ± 7.7	−13.8 ± 4.0*

The bolding is for emphasis of the differences and adds meaning to the understanding of the tests.

### 4.4 Effect of exposure on implicit associations

**Figure 3:**
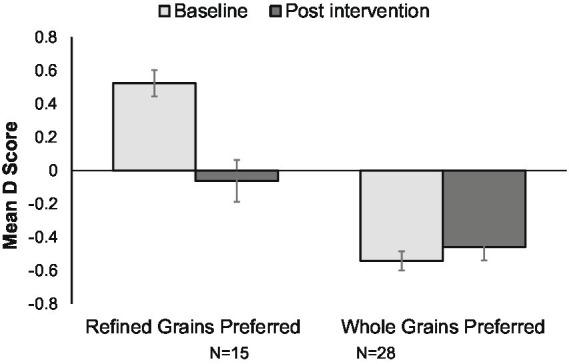
Implicit Preference for Grain Foods as Measured by D Score for all Participants. Bars represent mean IAT D scores with standard error bars. An implicit preference for refined grains is indicated by positive scores. At baseline, 15 of the 43 subjects preferred refined grains (left bars); at the end of intervention this preference changed (p<0.001) such that there was no clear preference for refined or whole grains. An implicit preference for whole grains is indicated by negative scores. At baseline, 28 of the 43 subjects preferred whole grains (right bars); this preference did not change at the end of intervention.

Another aim of this study was to determine if by simply providing WG foods to low WG consumers, thereby creating a mere exposure effect, implicit attitudes toward WG could be changed. The IAT *D* scores at baseline and week 6 were summarized for both groups to determine if exposure to the whole or refined grains changed the strength of association of WG foods with good taste. Controlling for initial *D* score, the change in *D* score was different between the WG and the RG intervention groups, (*p* = 0.034). There was no change in *D* score at 6 weeks in the WG intervention group, whereas the RG intervention group had a significant decrease in the *D* score, indicating a preference for whole grains (toward WG preference) (Figure 3).

**Figure 4:**
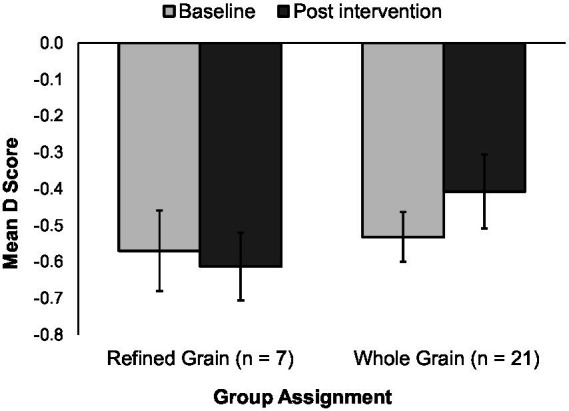
D scores in the 28 participants initially preferring whole grains, illustrated for groups receiving either refined grain products (left set of bars) or whole grain products (right set of bars). Bars represent mean IAT D scores with standard error bars. An implicit preference for whole grains is indicated by negative scores—responding more quickly in trials when whole grain food images were paired with positive taste descriptors and refined grain food images were paired with negative taste descriptors.

**Figure 5:**
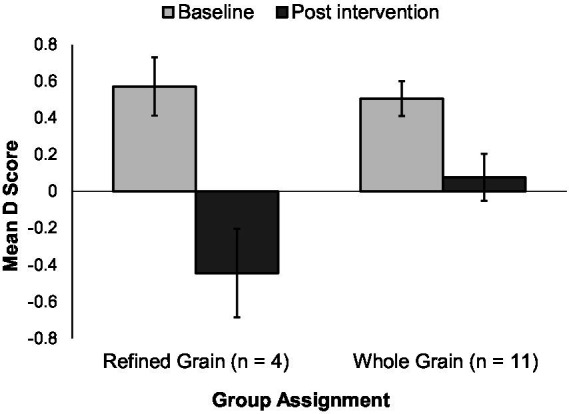
D scores in the 15 participants initially preferring refined grains. Bars represent mean IAT D scores with standard error bars. Positive scores indicate an implicit preference for refined grains and negatives scores indicate an implicit preference for whole grains.

For participants who initially preferred WG, this preference did not change regardless of whether they received the RG or the WG products (Figure 4). For participants who initially preferred RG, there was a significant shift away from preferring RG and toward preferring WG at week 6. This shift was apparent in the group receiving RG foods (*p* = 0.004) and also occurred in the group receiving WG foods (*p* = 0.032) (Figure 5).

## 5 Discussion

To our knowledge, this is the first study to utilize the Implicit Association Test as both a predictor and an outcome of mere exposure to a prolonged dietary intervention in a free-living setting. The Implicit Association Test measures the strength of association between a target concept and attribute; an implicit association is a non-conscious result of past experience which has been internalized and is not available to conscious introspection. Implicit measures may have better predictive validity regarding food choice than explicit measures, especially in situations where cognitive or emotional resources are limited due to stress or other factors (28–30). In this study, we aimed to see if an implicit association between refined or whole grain foods and taste would predict consumption of provided WG foods in low WG consumers in a free-living situation. We questioned if by merely exposing participants to WG, thereby creating new experience, would participants’ implicit association between those foods and taste be altered. Specifically, would mere exposure to WG result in a more positive *implicit* association between WG and taste? Furthermore, if the implicit association between WG and good taste was strengthened, would this result in increased consumption of those foods?

Our results were unexpected and intriguing. First of all, despite being self-identified low WG consumers, the majority of participants had a stronger implicit association of WG foods with pleasant taste than RG foods with pleasant taste before the intervention, based on their baseline IAT test scores. At no point during the screening process for the study did we ask participants whether they preferred refined or whole grain foods. Prospective participants were simply informed that this was a study “evaluating liking, acceptability, and health benefits of grain products.” The words “whole grain” and “refined grain” were never spoken to participants, and as they entered the study and received their study foods individually, participants were not aware that some received different products than they did. We did not want to bias their consumption of provided products by drawing attention to the perceived healthiness of the foods. Studies by Raghunathan et al. (38) demonstrated that for some individuals, foods perceived as ‘healthy’ are automatically associated with not being tasty, and this could have negatively affected consumption of provided study WG foods in the current study. Although we did not employ an explicit measure of preference for whole or refined grain foods, we thought it likely that low WG consumption would at least in part be due to a greater preference for the taste of RG foods over WG foods. Despite research indicating that the bitter taste of WG foods can negatively influence acceptability and consumption of WG products (57), the results of our initial Implicit Association Test suggest otherwise.

In answer to the first question, to determine if initial implicit associations between WG foods and taste would predict consumption of provided WG products, the answer was that it did not. Our results showed no difference in consumption of provided WG foods based on initial preference for whole or refined grains, as based on baseline participant IAT scores. In other words, even individuals with a more positive initial implicit attitude toward RG consumed the provided WG foods. These results are not entirely surprising given that the literature on the ability of the IAT to predict behavioral food choices has been mixed. Perugini (36) compared the predictive validity of implicit attitudes with that of explicit attitudes toward fruits versus snack foods and found that implicit attitudes, as measured by an IAT, better predicted a behavioral choice between a free piece of fruit or a snack (36). In contrast, Karpinski and Hilton (58) found that while both the IAT and explicit attitudes toward apples and candy bars showed the same preference, only the explicit attitude predicted behavioral choice. A number of different explanations for these discrepancies has been suggested. Ayres et al. (59) expanded on previous research and found that perceived palatability of food may influence the prediction of food choice beyond implicit measures. Meissner et al. (60) further explained how extraneous influences, such as task recoding, can affect the validity of the IAT and other implicit measures in predicting behavioral outcomes. That being taken into consideration, our results showing consumption of WG products in self-reported low WG consumers in a free-living setting suggest that switching RG for WG products is behaviorally feasible with little thought or effort.

In this study, mere exposure to WG in self-reported low WG consumers did not lead to a greater implicit association of WG with good taste or an increase in WG consumption over the course of the study. It is possible that the ability to change the implicit preference for WG or to increase WG consumption was hampered by the fact that the majority of participants implicitly associated WG with good taste at the start of the study. As the initial *D* scores were already indicative of a positive association, there may have remained little room for a measurable change in the positive direction (i.e., a ceiling effect). Future research with a longer-term exposure period to WG and RG foods may provide different outcomes with a more measurable change in preference and consumption of WG by individuals who self-identify as low WG consumers.

In participants who initially implicitly preferred RG foods and who were assigned the RG products, consumption of those foods declined over the 6-week intervention, whereas consumption of provided RG foods did not change over time in those who indicated a preference for WG foods initially. Considering all participants, the implicit association of RG foods with good taste declined between baseline and week 6. This shift occurred both in participants assigned RG and WG, although the shift was greater in individuals given RG products.

Our results showing decreased consumption of RG foods support the findings of Reynor and colleagues (61) who reported a ‘monotony effect’, which is defined as different from ‘sensory specific satiety’ (62). Sensory specific satiety refers to a phenomenon occurring within or shortly after a meal, tending to be of short duration, and the monotony effect refers to the decrease in perceived pleasantness of foods resulting from tasting the same flavors over time. The monotony effect tends to be of longer duration than that of sensory specific satiety (62). In their experiments Raynor and colleagues (63) found that by reducing dietary variety, consumption of provided food groups decreased. In the present study, it is possible that by providing self-reported RG consumers with an abundance of RG foods, we stimulated the monotony effect which resulted in decreased consumption of provided RG food and a greater implicit association of WG with pleasant taste. Zandstra et al. (64) found that when participants were allowed the greatest variety of choice in a study of product acceptance and in-home consumption of a meat sauce consumed once a week for 10 weeks, boredom ratings were lowest and acceptance rating highest in the group that was afforded the greatest variety. Given that the participants in the RG intervention group were presumably already consuming RG versions of provided study foods, this might explain the greater implicit association of images of WG grain foods and pleasant taste in the IAT task. However, given the small size of the group receiving the RG products, these findings should be considered with caution. In addition to reduced likelihood of detecting true effects, small sample sizes may conversely increase the odds of statistically significant results that are actually spurious.

It should be noted that the consumption of provided grain products averaged over the 6-week period was not different between intervention groups, with both groups consuming slightly less than half of grains servings provided. While participants were not restricted to consuming only the grain foods provided by the study, they were instructed to log all grain products in their weekly log booklets. The analyses we conducted were based only on the study foods because we could measure disappearance and check it against the log booklets. It would be informative to follow up this study with one where the non-study foods could also be included in the analyses, but this would require a method to accurately log outside foods. Advances in technology, such as smart phones with cameras to snap photographs of meals, may present an avenue for better documentation of food consumption in free living situations, not only for WG intake, but for all foods.

Whereas the present analysis is concerned with implicit processes that may be influenced by exposure to WG and concomitant effects on consumption of provided WG products, we have previously reported on health-related parameters associated with increased WG consumption in low WG consumers (52). We have also reported on the results of standard sensory evaluation testing of whole and refined grain foods before and after a 6-week exposure period to either WG or RG (53). We aimed to ascertain the specific sensory attributes of WG foods (e.g., overall liking, appearance, flavor, texture) that contribute to the willingness to include them in the regular diet. In addition, we investigated changes in implicit and explicit liking and wanting for other foods varying in fat content (high/low) and taste (sweet/savory) as result of exposure to WG as a potential health halo effect (53). Taken together, our published results contribute to understanding factors that lead to acceptance and liking of WG foods in addition to measuring clinical and physiological changes resulting from increased WG consumption.

Strengths of this study include the use of an implicit measure, the IAT, to investigate the influence of automatic attitudes on WG consumption in a free-living situation. Behavioral outcomes of nutrition interventions utilizing implicit measures in laboratory settings may not be generalizable outside of those settings. Additionally, using both disappearance and recorded consumption data allowed us to ascertain the effect of mere exposure to WG foods on implicit attitudes in healthy adults who were self-reported low WG consumers. By supplying participants with WG products in above the recommended amounts, we eliminated some of the commonly cited barriers to WG consumption, including cost, availability, and ability to identify WG at the point of purchase. Although poor taste has been reported to be the largest barrier to WG consumption, results from this study suggest that other barriers, such as texture, appearance, or cultural factors may play a more important role. This research contributes to the existing knowledge regarding barriers and facilitators to WG consumption and suggests further questions to investigate for increasing consumption of this important food group.

Limitations of this study include the inability to directly observe consumption of provided grain products. Participants were instructed to record intake of grain foods in provided log booklets, leaving data collection vulnerable to self-report bias. Although the free-living nature of the intervention was intended to deliver a more realistic picture of grain consumption, this entailed the inability to control for sharing of provided foods with others, simply discarding provided foods, or the addition of other grain products from outside sources. Future studies could be improved by including a validated physiological biomarker of whole grain consumption, such as plasma or urinary alkyl resorcinols to separate results from compliers and non-compliers in the outcomes of interest (65–67). A serious limitation to the present study was the overall small sample size and the unevenness of group sizes, which limited our power to detect group differences. Furthermore, in studies aimed at assessing barriers to WG consumption using implicit measures, the addition of an explicit measure of preference for one grain type over the other could provide information about which type of measure, implicit or explicit, better predicts WG consumption in a free-living situation. Previous studies have compared predictive validity of implicit and explicit measures in a variety of laboratory settings, which may not be indicative of behavior in real world settings (39, 42, 68, 69).

## 6 Conclusion

In this study, we aimed to determine the effects of implicit associations between whole and refined grains and taste and the effect of mere exposure on implicit associations and on consumption of provided grain foods. Initial preference for WG or RG foods did not predict consumption of WG products; consumption of provided WG foods did not differ between those who initially preferred refined or whole grains. Interestingly, we found that individuals who initially preferred RG foods decreased consumption of provided RG foods but not of provided WG foods. We questioned whether by mere exposure, implicit associations between WG foods and good taste would increase, which did happen, but only in the group initially preferring RG. On the other hand, implicit preference for RG decreased after 6 weeks of exposure to both refined and whole grains groups. This suggests that mechanisms other than initial taste preference may be at the root of choosing RG over WG. Future research into ways to replace some RG with WG, rather than focusing solely on increasing WG consumption, may represent an alternative strategy to support the Dietary Guidelines for Americans recommendations for grain intake to achieve health benefits.

## Data Availability

The raw data supporting the conclusions of this article will be made available by the authors, without undue reservation.
