# Supplementary material for: Initial implicit association between whole grains and taste does not predict consumption of whole grains in low-whole grain consumers: a pilot randomized controlled trial
Source: Front Nutr. 2024 Sep 30;11:1408256. doi: 10.3389/fnut.2024.1408256 (PMC11471685; doi:10.3389/fnut.2024.1408256)
Supplement: Supplementary file 1 [file Table_1.pdf]

Supplemental Table

Stimuli Used in the Implicit Association Test (IAT)

Refined Grain Foods	Whole Grain Foods	Good Taste	Bad Taste
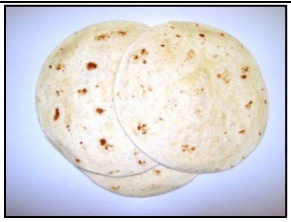	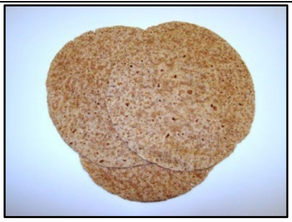	Tasty<br>Delicious<br>Yummy<br>Appetizing<br>Flavorful<br>Appealing<br>Mouthwatering	Disliked<br>Less tasty<br>Unappealing<br>Bland<br>Flavorless<br>Unappetizing<br>Unpalatable
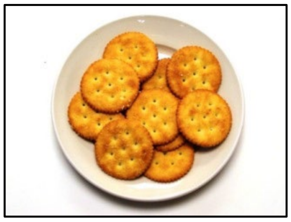	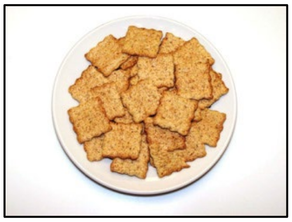		
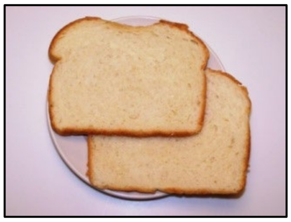	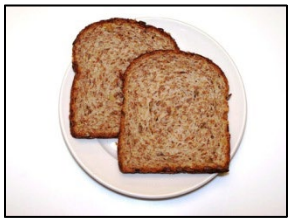		
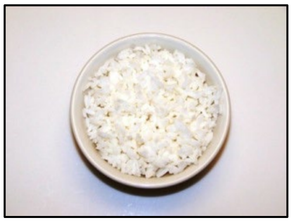	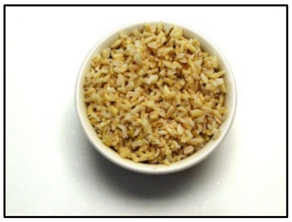		
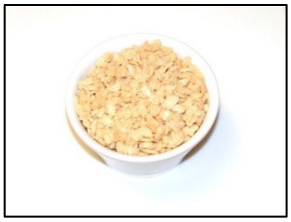	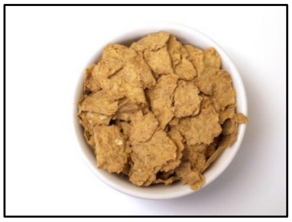		
