# Supplementary material for: Initial implicit association between whole grains and taste does not predict consumption of whole grains in low-whole grain consumers: a pilot randomized controlled trial
Source: Front Nutr. 2024 Sep 30;11:1408256. doi: 10.3389/fnut.2024.1408256 (PMC11471685; doi:10.3389/fnut.2024.1408256)

Supplemental Figure

Screenshots of Grains IAT demonstrating difference in screen position of target attributes Whole Grain and Refined Grain in the different test blocks

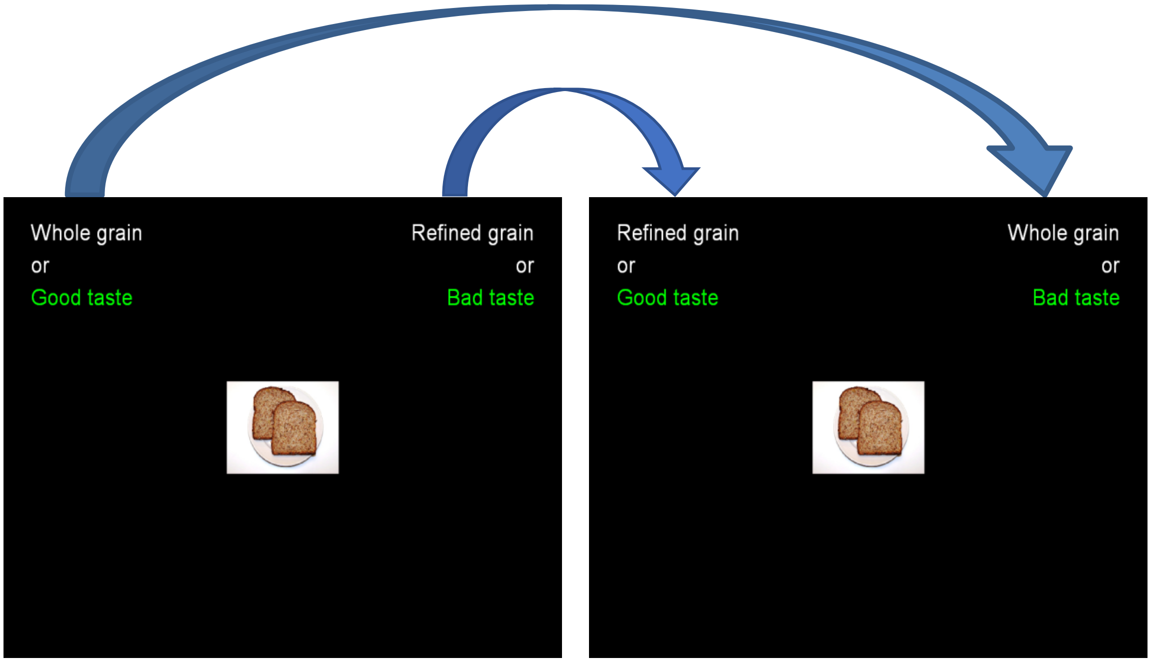

Supplement: Supplementary file 2 [file Image_1.pdf]
